# The influence of clinical risk factors on the classification of human cancer-associated fibroblasts in PDAC and pancreatitis patients

**DOI:** 10.1038/s44276-025-00150-5

**Published:** 2025-06-16

**Authors:** Viktoria Boeker, Lena Wilke, Ana Mansourkiaei, Van Manh H. Le, Kaira A. Church, Zoltan Czigany, Bo Kong, Fernanda G. Kugeratski, Jörg Kleeff, Jürgen Weitz, Christoph Kahlert

**Affiliations:** 1 Department of Visceral, Thoracic and Vascular Surgery, University Medical Center Carl Gustav Carus Dresden, Dresden, Germany; 2 TUD Dresden University of Technology, Dresden, Germany; 3 Department of General-, Visceral-, and Transplant Surgery, Ruprecht Karl University Heidelberg, University Medical Center Heidelberg, Heidelberg, Germany; 4 Department of Cancer Biology, University of Texas MD Anderson Cancer Center, Houston, USA; 5 Department of Experimental Therapeutics, University of Texas MD Anderson Cancer Center, Houston, USA; 6 Department of Visceral, Vascular and Endocrine Surgery, Martin-Luther-University Halle-Wittenberg, University Medical Center Halle, Halle, Germany

## Abstract

Cancer-associated fibroblasts (CAFs) constitute an important cell population in the microenvironment of pancreatic cancer. They can arise from disease-associated fibroblasts (DAFs) to support or restrain tumor growth. How many CAF subtypes exist and what signals drive their development is unclear. Currently, there are three commonly accepted subtypes, namely myofibroblast-like (myCAF), immunomodulatory (iCAF), and antigen-presenting (apCAF). Here, we analyzed the correlation between clinical risk factors with the proportion of each CAF subtype. In our patient cohort (*n* = 21), we investigated DAFs from patients with chronic pancreatitis (CP) and CAFs from pancreatic ductal adenocarcinoma (PDAC) patients after surgical resection via flow cytometry and RNA expression analysis. The expression of iCAF marker Interleukin-6 displayed significant differences depending on lifestyle factors, such as smoking status, age, and Body Mass Index (BMI). The apCAF marker HLA-DQA1 correlated with age. The largest difference showed the quantitative difference of apCAF markers in ~40% of PDAC- and ~20% of CP patients. In conclusion, clinical risk factors may influence the prevelance of specific CAF subsets. Unraveling the complex interplay between CAFs and tumor cells is crucial for novel therapies to improve long-term survival for pancreatic cancer patients.

## Background

Pancreatic cancer is a life-threatening disease feared by people worldwide. After diagnosis with pancreatic ductal adenocarcinoma (PDAC), the probability of death within 5 years is 88% [1].

Despite tremendous research advances, PDAC is predicted to become the second deadliest cancer in the next two decades [2]. Due to the aging society and its association with the Western lifestyle, cases of people diagnosed with PDAC are increasing [3]. The lack of biomarkers and early diagnostic tools for this mostly asymptomatic cancer leads to diagnosis in advanced stages that present with metastasis [1]. Ultimately, the majority of patients is not eligible for surgery, which is the only curative treatment up to date [3]. Consequently, breakthrough targets and therapies to fight this malignancy are crucial.

**Fig. 1:**
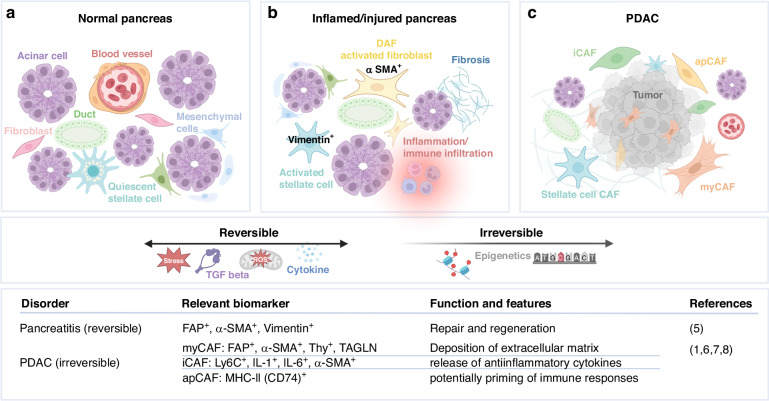
Fibroblasts in the normal pancreatic microenvironment, during activation and transition to cancer-associated fibroblasts. In the healthy pancreatic microenvironment, fibroblasts and their subgroups like pancreatic stellate cells are, e.g., responsible for storing lipids and vitamin A (**a**). Due to factors like stress, ROS, and others, they get reversibly activated as disease-associated fibroblasts (DAFs) ${{\rm{\alpha }}}$-SMA^+^, FAP^+^, Vimentin^+^ (**b**). Functionally, they repair and regenerate injured tissue. After further epigenetic activation, they can irreversibly transition to cancer-associated fibroblasts. The most common subgroups are myCAFs: FAP^+^, ${{\rm{\alpha }}}$-SMA^+^, Thy^+^, TAGLN^+^, iCAFs: Ly6C^+^, IL-1^+^, IL-6^+^, ${{\rm{\alpha }}}$-SMA^+^ and apCAFs: MHC-II (CD74) ^+^ (**c**). Created with BioRender.com

PDAC tumor microenvironment (TME) has come to the forefront, with a dense stroma and a lack of functional vasculature, leading to a hypoxic environment and reduced immune cell infiltration [4], which are described as ‘cold tumors’ [1]. A centerpiece in the challenging puzzle of how TME and tumor cells operate are cancer-associated fibroblasts (CAFs) [5]. Some CAFs' promote tumor growth, and some restrain it. Potentially, a feature is that they limit tumor progression via matrix protein deposition [4]. Other CAFs can secrete metabolites to support tumor growth or suppress anti-tumor immunity [4]. Studies suggest that after activation, fibroblasts differentiate into a reversible state where they are ${{\rm{\alpha }}}$-smooth muscle actin $({{\rm{\alpha }}}{{\rm{SMA}}})$ and vimentin-positive (Fig. 1b) [6]. CAF activation is due to factors such as stress, reactive oxygen species (ROS), transforming growth factor beta (TGF-${{\rm{\beta }}}$) or cytokines [7]. In a situation of tissue damage like in pancreatitis, activated fibroblasts produce collagen aiming to repair and regenerate damaged stromal tissue [8–10]. In this stage, they can be called disease-associated fibroblasts (DAFs) [11]. Further epigenetic influences can cause these activated fibroblasts to irreversibly transition into CAFs, influencing pancreatic cancer growth (Fig. 1c) [6].

In PDAC, CAFs have been categorized into different subsets called immunomodulatory CAFs (iCAFs), myofibroblast-like CAFS (myCAFs), and antigen-presenting CAFs (apCAFs) (Fig. 1c) [12–15]. Up to 50% of the CAF population are myCAFs, primarily contributing to the extracellular matrix and located near the tumor cells [16–18]. Whereas iCAFs are prone to filling gaps between nests of tumor cells and releasing inflammatory cytokines such as interleukins IL-1 and IL-6 [1, 6, 13]. The smallest subgroup comprises apCAFs whose attributed functions resemble myeloid populations in the antigen-presenting process [19, 20]. Further they are involved in the promotion of immunosuppression by expression of CD74 [15].

A potential plasticity between myCAFs and iCAFs in PDAC has been suggested [21]. Furthermore, secretion of IL-6 and through ${{\rm{\alpha }}}$-SMA resistance against first-line chemotherapy gemcitabine is promoted [22, 23]. The contribution of CAFs’ to the epithelial-mesenchymal transition (EMT) amongst their production of hepatocyte growth factor (HGF) is one way of driving resistance [24]. The mTOR/4E-PB1 pathway also plays a key role in addition to the stromal cell-derived factor 1 (SDF-1), which is secreted by CAFs [23, 25]. Despite the secretion of various factors, CAFs build up a physical barrier via stroma production, which causes vascular collapse, impeding chemotherapy from reaching tumor cells [26, 27]. Drugs like a PEGylated human recombinant PH20 hyaluronidase (PEGPH20) could reverse this issue through the reduction of hyaluronic acid (HA), leading to increased delivery of gemcitabine in PDAC [28]. Nonetheless, no clinical drugs effectively can prevent CAFs’ IL-6 secretion yet to prolong survival. Underscoring once more the importance of studies on targeting and classifying CAFs. Even if the transition between the multiplex subgroups are analyzed via lineage tracing, detailed key factors influencing these phenomena remain unclear [21].

This study investigated the expression of markers defining the three main CAF subsets in patients with pancreatitis and pancreatic cancers. Furthermore, the contribution of important clinical risk factors (namely gender, age, diabetes type 2, smoking, body mass index (BMI), and tumor stage) in modulating the abundance of different CAF subsets was evaluated.

## Material and methods

This study investigated pancreatic tissues from 21 patients who underwent surgery at the University Hospital Carl Gustav Carus Dresden and the University Hospital Halle (Ethical approval EK 76032013 Dresden, Ethical approval 037 Halle). All patients provided written consent according to the Declaration of Helsinki.

Tissues of 15 diagnosed pancreatic cancer patients, four diagnosed chronic pancreatitis patients, and two tissue samples of patients in pre-disease stages were analyzed.

All patient data were fully anonymized in accordance with the ethical protocol of the Surgical Laboratory of the University Hospital Dresden and securely stored to ensure confidentiality and compliance with data protection standards.

### Primary cell culture

Disease-associated fibroblasts and cancer-associated fibroblasts were isolated from the tissues using the outgrowth method [29]. Under sterile conditions, tumors, pancreatitis, and pre-disease tissues were cut into pieces of 3–5 mm^2^. CAF-specific media components included Ham’s F12 Medium (Gibco, UK), low glucose Dulbecco’s Modified Eagle’s Medium (DMEM) (1X, Gibco, UK), fetal bovine serum (FBS) (Gibco, UK), amphotericin B (250 μg/ml, Sigma-Aldrich, USA) and penicillin/streptomycin (10.000 Units penicillin +10 mg streptomycin per ml in 0.9% NaCl, Sigma-Aldrich, USA). Tissues were cultured with 5–7 ml of media and incubated at 37 °C and 5% CO_2_. On an average of 10 days post-surgery, cells were transferred from 10 × 15 mm Petri dishes to 75 cm^2^ flasks and cultured until confluency of 90% for further experiments.

### RNA Isolation and qPCR

RNA was isolated (in passage 2) according to the manufacturer’s protocol using the RNeasy Mini Kit (Qiagen, Netherlands). After quantification by NanoVue Plus spectrophotometer (GE Healthcare Technologies, USA), qPCR analysis was performed using the StepOne Real-Time PCR System (Thermo Scientific, USA). 1000 ng of RNA from each sample was transcribed into cDNA. The enzyme was mixed with 4.8 µl of MasterMix and brought to a volume of 20 µl with DNase/RNase-free water (Fisher Scientific, USA). A MasterMix contained 2.0 µl of 10× RT Buffer, 2.0 µl of 10× RT Random Primers, 0.8 µl of 25x dNTP Mix, and 1.0 µl of reverse transcriptase. To run the RT-qPCR SYBR Green qPCR Master Mix (Life Technologies, USA), primers were obtained from Eurofins Genomics, Germany (see Table 1).

**Table 1:** List of the used primer and their sequences.

Primers	Sequence (5‘→3´)
FAP forward	AGA ATT CAA CTG TGA TGG CA
FAP reverse	GTG CAC ATT ATC ATC TGC TG
HLA-DQA1 forward	GCT ACC AAT GAG GTT CCT G
HLA-DQA1 Reverse	AAG ACA AAT GAG GGT GTT GG
PTPRC forward	TAT TTG TGA CAG GGC AAA GC
PTPRC Reverse	CAC TTG AAA GTG GAA CAC TGG
EPCAM forward	CGA GTG AGA ACC TAC TGG A
EPCAM Reverse	TGA TCT CCT TCT GAA GTG CA
ACTA2 forward	CAT CGG AAA TGA AAT GAA CGT TTC C
ACTA2 Reverse	CCC CTG ATA GGA CAT TGT TAG C
IL6 Forward	TGG CTG AAA AAG ATT GGA TGC T
IL6 Reverse	AAC TCC AAA AGA CCA GTG ATG ATT T
Vimentin forward	GGA CCA GCT AAC CAA CGA CA
Vimentin Reverse	AAG GTC TCC TCC TGA TGG TG
PolR2A forward	CGC ATT GAC TTG CGT TTC CA
PolR2A Reverse	TTT TGT GCA GAG TTG GCT GC
RPL32 forward	CCA CCG TCC CTT CTC TCT TC
RPL32 Reverse	GCT TCA CAA GGG GTC TGA GG

### Flow cytometry

Cell pellets (0.2 × 10^6^ cells) were resuspended in 100 µl of FACS buffer (PBS with 2% FBS). After blocking with FcR Blocking Reagent in a concentration of 1:50, the mixture was incubated for 20 min at 4 °C in the dark. The solution was removed after centrifugation, and the pellet was resuspended and incubated with 5 µl of the antibodies (CD45, CD326, FAP alpha, Ly6G6D, HLA-DP, CD74) and 1 µl of the IL1 -R1 antibody. Samples were incubated for 30 min at 4 °C, protected from light. After two washes with FACS buffer, samples were resuspended in 500 µl of FACS buffer. After staining the cells with the antibodies (see Table 2), cells were analyzed by flow cytometry in the core facility of the University Hospital Dresden using the BD LSR Fortessa Cell Analyzer (BD Biosciences, USA).

**Table 2:** List of the used antibodies for flow cytometry.

Antibody	Clone	Fluorochrome	Vendor	Catalog number
CD45	I3/2.3	Alexa Fluor 488	BD Biosciences, USA	567402
CD326	9C4	Alexa Fluor 488	BioLegend, USA	324209
Fibroblast activation protein α	#427819	Alexa Fluor 594	R&D Systems, USA	FAB3715T
IL-1 R1	Polyclonal	Alexa Fluor 647	VWR International, USA	BS-5394R-A647
Ly6G6D	13.8	PE	BioLegend, USA	367003
HLA-DP	B7/21	BV711	BD Biosciences, USA	750870
CD74	LN2	BV605	BD Biosciences, USA	743733
Isotype control: IgG1 κ	MOPC-21	PE/Dazzle 594	BioLegend, USA	400176

### Immunocytochemistry

Immunocytochemistry (ICC) on DAFs and CAFs was performed with antibodies specific for fibroblast activation protein (FAP), vimentin, and alpha-smooth muscle actin (Table 3). Shortly, 3 × 10^4^ and 4 × 10^4^ cells were seeded in chamber slides with 500 ml medium per chamber and incubated for 48h. Afterward, cells were fixed using a 4% formalin solution (SAV Liquid Production GmbH, Germany). After two washing steps with PBS, slides were incubated in 50 mM ammonium chloride solution (Merck, Germany) for 10 min to quench the fixative. Additionally, two PBS washes were conducted, and cells were incubated with 0.2% Triton-X 100 (Merck, Germany) for 15 min for permeabilization. Methanol and 3% H_2_O_2_ (Carl Roth, Germany) were incubated as a blocking solution for 20 min at 4 °C. After two washes with PBSB (0.1% BSA (Carl Roth, Germany) in 50 ml PBS) for 5 min, the Universal blocking reagent (BioGenex, USA) was added in a 1:10 solution and incubated for 30 min protected from light. Slides were then incubated with primary antibodies (Table 3) overnight in the dark at 4 °C. Antibody diluent from Dako (USA) was employed. As a negative control, cells (without primary antibody) were only incubated with the antibody diluent. The next day, cells were washed 3 × 5 min (2× PBSBT (0.1% BSA and 0.05% Tween 20 in PBS), 1× PBSB), and 200 ml per chamber of the secondary antibody was added and incubated for 60 min, protected from light. Immunostainings were visualized with 3’3’-Diaminobenzidin-Tetrahydrochlorid (DAB) (Dako, USA). For nuclei staining, the slides were incubated with Haemalaun (Merck, Germany) for 20sec and cleaned under running water for 10 min. After fixation via Permount Mounting medium (Biolyst, USA), the microscope BZ-X810 (Keyence, Germany) was used for imaging.

**Table 3:** List of the used antibodies for immunocytochemistry.

Antibody	Vendor	Catalog number	Dilution
α-smooth muscle actin	Abcam, UK	ab5694	1:200
Vimentin	Abcam, UK	Ab92547	1:1000
Fibroblast activation protein α	Abcam, UK	ab207178	1:500
HRP labeled polymer anti-rabbit	Agilent Technologies, USA	K4003	–

For the quantitative analysis of stained cells, the slides were scanned using the BZ-X810 fluorescence microscope at constant exposure time and intensity, with magnifications of 4× and 20×. The resulting image data were subsequently analyzed using ImageJ software. For each patient, two biological replicates were used. Then, the 4× magnification images were loaded into the software. The images were converted into 8-bit grayscale images through a plug-in, and a uniform threshold value was defined for each antibody. This allowed the calculation of the area fraction exhibiting a specific staining intensity, enabling quantification of the cells stained by each antibody. This analysis was performed on slides with 3 × 10^4^ and 4 × 10^4^ cells per chamber and normalized to 10,000 cells per chamber. Finally, the arithmetic mean of the two results was calculated to obtain a single result for each patient sample.

### TCGA analysis

The Pancreatic Adenocarcinoma (TCGA, PanCancer Atlas) cohort was analyzed using cBioPortal [30]. The cohort was segregated into two groups based on the median expression of IL-6, as determined by mRNA expression z-scores relative to diploid samples. Probability of survival and clinical features of patients within IL-6 high (*n* = 89) and IL-6 low (*n* = 88) groups were downloaded from cBioPortal.

### Statistical analysis

All statistical analyses were performed using Graph Pad Prism version 10. Normal distribution was evaluated with Shapiro–Wilk Test. Unpaired t-tests were applied depending on experimental results. Otherwise, Kolmogorov–Smirnov tests were applied. A two-way ANOVA analysis was utilized for marker comparisons/classifications after flow cytometry. Ordinary one-way ANOVA was applied to quantify differences in tumor stage marker expressions in the case of regular distributions. Alternatively, the Kruskal–Wallis tests were performed. The statistical significance was established at *p* < 0.05. The significance limits are shown as follows: *p < 0.05; **p < 0.01; ***p < 0.001.

## Results

### Isolation of fibroblasts from 21 human pancreatic tissue samples

**Fig. 2:**
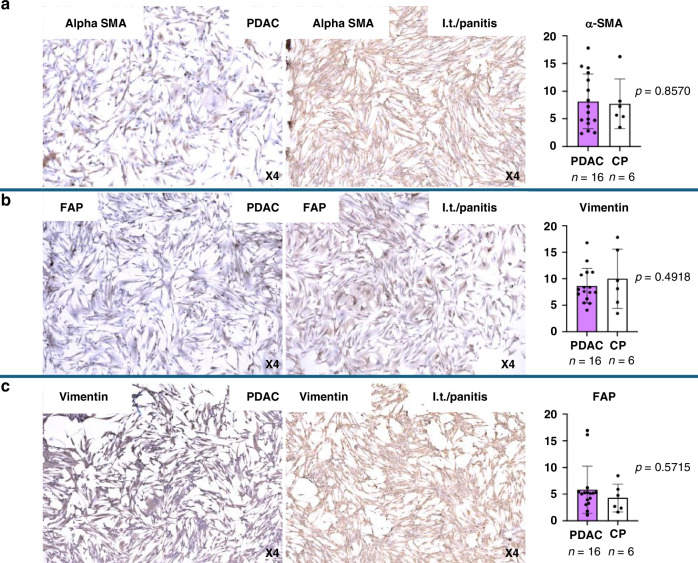
Immunocytochemistry (ICC) of DAFs and CAFs confirm α - SMA, FAP and Vimentin expressions in cells from PDAC and chronic pancreatitis patients. Representative staining displays cytoplasmatic α-SMA (**a**), FAP (**b**), and Vimentin (**c**). Expression levels of α-SMA (**a**), FAP (**b**), and Vimentin (**c**) are compared in CAFs of PDAC (*n* = 16) vs. DAFs of chronic pancreatitis/ pre-disease patients (*n* = 6). Magnification: x4.

Our study includes the investigation of tissue-derived human-activated disease-associated fibroblasts (here called DAFs) and cancer-associated fibroblasts (CAFs) of tissues from 15 patients who underwent surgery at the University Hospital of Dresden and University Hospital in Halle assured to be diagnosed with pancreatic ductal adenocarcinoma (PDAC). Additionally, DAFs were analyzed from post-surgery tissues of four patients diagnosed with chronic pancreatitis. In addition, DAFs from two tissues of patients in pre-disease stages were included in the non-cancer/inflamed tissue group. All tissue samples were cultured similarly to isolate/select DAFs and CAFs within two to three weeks using the outgrowth method. A typical spindle cell shape morphology was observed for all fibroblasts similarly (Fig. 2a–c). Overall, growth rates were also comparable across different samples. Finally, we confirmed the purity of fibroblasts with ICC staining for fibroblast markers (Fig. 2a–c).

### DAFs express various levels of Vimentin, CAFs express FAP and ${{\boldsymbol{\alpha }}}$-SMA

Previous single cell-based studies categorize fibroblasts into three subpopulations: myCAFs, iCAFs, and apCAFs [31].

Our study aims to focus on human PDAC tissues and samples from other pancreatic diseases like chronic pancreatitis to further investigate the differences and similarities between fibroblasts. This is intended to establish whether the full spectrum of DAF/CAF heterogeneity also applies to pancreatitis tissues.

Initially, the presence of Vimentin, ${{\rm{\alpha }}}$SMA, and Fibroblast Activation Protein (FAP) by immunocytochemistry staining confirmed the isolation of DAFs and CAFs (Fig. 2a–c). All samples were positive for these markers. Quantitative analysis shows a trend for higher prevalence of FAP^+^- (1–15%; +/−SEM 1.1%, *p* = 0.5715 for DAF vs. CAF) and ${{\rm{\alpha }}}$SMA^+^ CAFs (2–15%; +/−SEM 1.2%, *p* = 0.8570 for DAF vs CAF) in CAFs compared to pancreatitis DAFs/normal tissue samples (FAP 2–6%, +/−SEM 1%; ${{\rm{\alpha }}}$SMA 3–12%, +/−SEM 1.8%) (Fig. 2). According to our analysis, Vimentin is expressed in a broader range within DAFs of pancreatitis/pre-disease tissues (3–14%, +/−SEM 0.8%, *p* = −0.4918) compared to CAFs (4–12%, +/−SEM 2.2%) suggesting that Vimentin is more specific for fibroblasts in a reversible activated state before becoming a CAF. However, none of the data is statistically significant in the current cohort, which could be due to too-small sample size, or the markers might express, in general, a high heterogeneity.

### CAF subsets are heterogenous in abundance

**Fig. 3:**
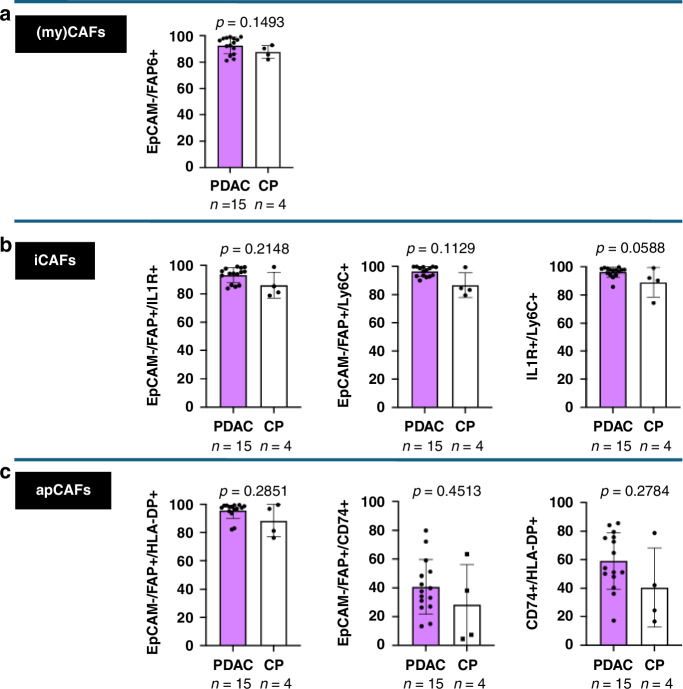
Flow cytometry analysis for fibroblast subtype classification. Percentages of EpCam^-^/ FAP^+^ fibroblasts in PDAC (*n* = 15) and CP (*n* = 4) samples (**a**). Percentages of EpCam^-^/FAP^+^/IL1R^+^ -, EpCam^-^/FAP^+^/Ly6C^+^-, and EpCam^-^/FAP^+^/IL1R^+^/Ly6C^+^ fibroblasts in PDAC (*n* = 15) and CP (*n* = 4) samples (**b**). EpCam^-^/FAP^+^/HLA-DP^+^-, EpCam^-^/FAP^+^/CD74^+^-, and EpCam^-^/FAP^+^/HLA-DP^+^/CD74^+^ fibroblasts in PDAC (*n* = 15) and CP (*n* = 4) samples (**c**).

We used flow cytometry analysis to examine DAF and CAF populations and their heterogeneity in human pancreatic diseases after isolating fibroblasts from 15 PDAC (pT1-3) - and four chronic pancreatitis with low-grade PanIN tissues. Viable cells were stained with DAPI, and EpCAM plus CD45 were applied as negative selection markers to exclude epithelial and immune cells, respectively (Supplementary Fig. 1). The majority of cells in our viable cell fraction were classified as EpCAM^-^/FAP^+^ positive cells. For CAFs, the mean FAP fequency was 93% (+/−SEM 1.6%), which is 5% higher than the DAFs from pancreatitis tissue, which expresses a mean of 88% FAP^+^ fibroblasts (+/−SEM 2.4%) (Fig. 3a). FAP is selective for cancer-associated fibroblasts in tumors and myCAFs display the largest fibroblast population. To further understand the composition of CAF subpopulations in the pancreatic microenvironment, gating was conducted from FAP^+^ fibroblasts to iCAFs and apCAFs (Supplementary Fig. 1). To define iCAFs, the markers Interleukin 1 Receptor 1 (IL1R) and Lymphocyte antigen 6 (Ly6C) were used. In cancer tissues, 93% (+/−SEM 1.3%) of the EpCAM^-^/FAP^+^ fibroblasts were also IL1R^+^ and 96% (+/−SEM 0.8%) were also Ly6C^+^ (Fig. 3b), suggesting a high feature of plasticity between myCAF and iCAF populations. For pancreatitis samples, 87% (+/−SEM 2.4%) were EpCAM^-^/FAP^+^; gating further results in 86% IL1R^+^ (+/−SEM 4.4%) and Ly6C^+^ (+/−SEM 4.3%) cells (Fig. 3b). This led to the finding that in inflamed pancreatitis tissue, the presence of iCAFs is 10% lower than in cancer tissues.

The two investigated cohorts corroborate previous studies that show that myCAFs and iCAFs represent the majority of CAF populations in diseased human pancreatic tissues [1, 17, 32, 33]. As a novel discovery, it can be concluded that CAF subgroups are also relevant in the microenvironment of pancreatitis tissue. Thus, functional investigations are crucially needed to detect the role of fibroblast subgroups in pancreatitis and ascertain the timepoint of irreversible action of CAFs.

### apCAFs are higher expressed in CAFs than DAFs

One of the latest detected subgroups of CAFs are the antigen-presenting CAFs. Elyada, Tuveson et al. discovered apCAFs in the widely used KPC mouse model and confirmed their existence in a small cohort of human PDAC tissues [19].

In our study, we analyzed 15 human pancreatic cancer tissues and four pancreatitis tissues via flow cytometry utilizing HLA-DP as a marker to define and reveal that CAFs in vitro without stimulus retain HLA receptors. CD74 was additionally used as an apCAF marker (Supplementary Fig. 1). On average, 96% (+/−SEM 1.4%) of the PDAC CAFs were HLA-DP^+^, and 88% (+/−SEM 5%) of the pancreatitis DAFs (Fig. 3c). However, in the cohort of 15 CAF samples, the expression of CD74^+^ cells showed substantially higher levels: in median ~40% (+/−SEM 4%) (Fig. 3c). Whereas for the DAFs in pancreatitis tissue, our results align with the prior findings of a median of ~20% CD74^+^ cells (+/−SEM 13%) (Fig. 3c). This supports that apCAFs play an important role in human pancreatic microenvironments, especially in cancers. Further investigations need to clarify, especially the functional role of CD74^+^ DAFs in pre-cancer diseases for a tumor-restraining or tumor-promoting role.

### The iCAF marker *IL*6 correlates with pancreatic cancer risk factors

**Fig. 4:**
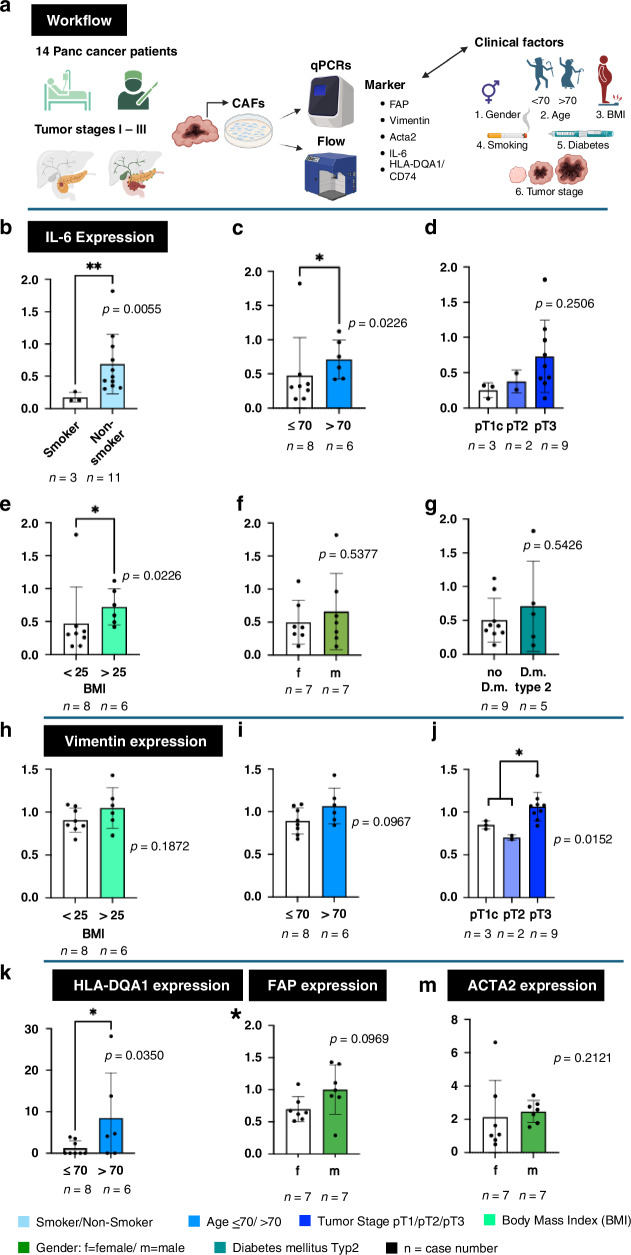
Correlation of clinical risk factors with the expression of CAF subset markers. Clinical risk factors for pancreatic diseases and tumor outcomes like Age, BMI, smoking, diabetes type2, and tumor stage were correlated with markers for the CAF subgroups myCAFs, iCAFs, and apCAFs (**a**). Expression levels of *IL-6* (**b**–**g**), *Vimentin* (**h**–**j**). *HLA-DQA1* (**k**), *FAP* (**l**), and *ACTA2* (**m**) are shown. Unpaired t-tests were applied depending on experimental results. Otherwise, Kolmogorov–Smirnov tests were applied. Created with BioRender.com.

To establish the importance of CAF heterogeneity in human tissues, the potential influences of clinical risk factors on DAF and CAF populations were examined (Fig. 4a).

This study includes gender differences (male vs. female), age (<70, >70), body mass index (BMI<25 = normal weight vs. BMI>25 = overweight), smoking, type 2 diabetes, and different tumor stages (pT1-pT3). Statistical analysis was applied after measuring the gene expressions via qPCR of the isolated RNA of all PDAC samples. The essential markers for the currently most common groups are for myCAFs defined by *Vimentin*, *ACTA2* for *αSMA* and *FAP*, iCAFs determined by the marker gene *IL6*, and apCAFs by *HLA-DQA1*.

The influence of risk factors on the expression of *IL6* (for iCAFs) was striking (Fig. 4b–g). Smoking is a significant risk factor correlating with the reduction of the expression of iCAFs (*p* = 0.0055, +/−SEM 1.4%) (Fig. 4b). At the same time, an older age (>70) causes enrichment of gene expression for iCAFs (*p* = 0.0226, +/−SEM 0.2%) (Fig. 4c). The same phenomenon was observed for obese patients with an increased expression for iCAF marker *IL6* compared to patients with a normal body weight (*p* = 0.0226, +/−SEM 0.2%) (Fig. 4e). Obesity is associated with type 2 diabetes mellitus. However, the correlation of the expression of *IL6* in patients with diabetes mellitus type 2 in the present sample size and composition was not statistically significantly higher than in non-diabetic patients (Fig. 4g). No significant relevance was observed in the investigated cohort for the gender analysis (males vs. females) or in the investigated tumor stages. However, a trend was observed toward higher *IL6* expression in advanced-stage tumors (Fig. 4d, f). This data suggests that iCAF populations are linked to clinical risk factors of high BMI, smoking, and age.

The myCAF marker *Vimentin* also impacts CAF heterogeneity (Fig. 4h–j). Patients in the population of a pT3 tumor stage show significantly higher *Vimentin* expression (*p* = 0.0152, +/−SEM 0.2%) (Fig. 4j). Age above 70 also shows a trend towards an increase in *Vimentin* expression (*p* = 0.0967, +/−SEM 0.1%) (Fig. 4i). Likewise, obesity is a factor that correlates with a trend for increased *Vimentin* expression (Fig. 4h). Patients above the age of 70 express significantly higher *HLA-DQA1* levels (*p* = 0.0350, +/−SEM 4%). For both markers *FAP* and *ACTA2*, we observed that males have a trend towards a higher *FAP* expression in CAFs compared to females and a trend towards a higher expression of *ACTA2* (Fig. 4l, m). Nonetheless, these trends were in the present cohort, and its composition not statistically significant. Also, type 2 diabetes, which is globally due to lifestyle as a risk factor for pancreatic diseases, has no significant influence on the DAF- and CAF population.

Overall, *IL6* is dominant in CAF populations, correlating with clinical risk factors. Our findings are highly relevant since further studies characterized *IL6* as a factor mediating resistance to gemcitabine [34]. Our investigations could provide a novel strategy to select patients more likely to be responsive to gemcitabine therapies. Respectively favorable to reduce *IL6* levels is a female sex, age under 70, and smoking. In contrast, a BMI >25, a pT3 tumor stage, and D.m. type 2 are potentially unfavorable factors increasing *IL6*.

### Impact of *IL-6* on survival and tumor neoplasm

**Fig. 5:**
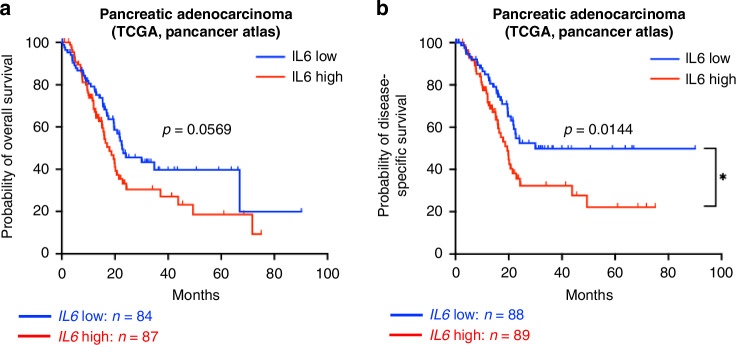
Survival analysis in patients expressing high- or low *IL6* after RNA sequencing of tumor bulk tissues. Kaplan–Meier plot showing Disease-specific Survival (*n* = 171) (**a**) and Overall Survival (*n* = 177) (**b**) of patients with PDAC having a low *IL6* or high *IL6* level in RNA of tumor bulk tissues. *P* values are as indicated in the respective graph.

Next, we used TCGA data to investigate whether *IL6* expression levels in pancreatic tumors influence survival. The pancreatic adenocarcinoma cohort was segregated into two groups based on the median expression of *IL6* (Fig. 5a). For these patients, the analysis of disease-specific survival revealed a significant difference, demonstrating that a low *IL6* expression is correlated with prolonged disease-free survival (*p* = 0.0144). Similarly, the overall survival of patients with low *IL6* expression favors prolonged survival, although the difference is not statistically significant compared to the group of patients with high *IL6* (Fig. 5b).

Looking further into clinical information within the *IL6* high and low cohorts, there was a trend towards 60% of the males having a low *IL6* and 40% of the females having low *IL6* (Supplementary Fig. 4). Further, there was a trend that a higher neoplasm histologic grade, a higher tumor stage code, and a higher neoplasm disease stage trend towards higher *IL6* expression (Supplementary Fig. 4).

In summary, the results of this study demonstrate the importance of including clinical risk factors in the categorization of the expression of fibroblast heterogeneity and complexity and, respectively, therapeutic decisions that needs further investigations.

## Discussion

This study investigated the heterogeneity and complexity of human DAFs and CAFs in 21 pancreatic tissues. CAFs from 15 patients diagnosed with PDAC and 6 DAF samples from patients with pancreatitis and pre-cancer diseases were evaluated. Former studies were primarily based on genetically engineered mouse models, which cannot always accurately reflect what is observed in human patient samples [35]. Despite tremendous advances throughout the last decade, there is still no widespread consensus on an fibroblast classification and function in the tumor microenvironment [31]. Employing a novel classification strategy, including clinical risk factors to understand the complexity and heterogeneity of this cell group, serves to investigate the influence of fibroblast distribution.

A recently published study by Lee et al. investigated via RNA sequencing on resected PDAC tumors if so-called classical CAFs or resting CAFs influence patients' overall survival [34]. They found that resting CAFs lead to a better outcome. Furthermore, they compared additional neoadjuvant chemotherapy with FOLFIRINOX to determine if it also influences the outcomes depending on the CAF type. This resulted in the finding that patients who received neoadjuvant chemotherapy have a higher expression of resting CAFs and obey the best survival. The group concludes that not only tumors but also CAFs should undergo molecular subtyping for the selection of patients' therapy regimes [34].

Fibroblasts cultured in 2D models show that mainly all cells express the activated myCAF phenotype. However, *IL6*^+^ iCAFs especially exemplify an impressive influence in expression depending on clinical risk factors like smoking, age, and BMI. Substantiating once more the dynamics in CAF's nature to interconvert between subtypes here influenced by clinical risk factors.

Previous studies found that different secreted factors, such as IL-6 by CAFs and other pathways, can influence gemcitabine resistance in PDAC [36].

Another work by Zhang et al. showed that IL-6 promotes PDAC progression by promoting oxidative stress resistance and MAPK signaling activation [37]. According to their study, the absence of IL-6 leads to the clearance of precursor lesions in pancreatic tissues based on mechanisms involving epithelial-mesenchymal interactions.

Supporting the thesis that high IL-6 baseline levels imply a poorer outcome was concluded after a Phase II clinical trial by Dorff et al. in which Siltuximab, a Monoclonal Antibody against Interleukin 6 in pre-treated patients with prostate cancer, was applied [38].

In the current ongoing phase II study, the IL-6 inhibitor Tocilizumab in combination with Ipilimumab and Nivolumab is investigated in patients with Stage III or Stage IV Melanoma (NCT03999749). Parallel in another study, also for patients with Stage III or Stage IV Melanoma, the IL-6 Receptor Inhibitor Sarilumab is tested (NCT 05428007).

Lastly, another trial investigates in Phase Ib/II the effects of Siltuximab and Spartalizumab in patients with metastatic pancreatic cancer (NCT04191421 [39]). In this trial, the authors aim to reduce the chimeric monoclonal antibody that specifically targets IL-6 molecules in the immunosuppressive tumor environment.

Due to our findings, a hypothesis is that patients could influence unfavorable high levels of *IL6* positive iCAFs by decreasing body weight. However, the factor of smoking to reduce iCAFs needs to be carefully evaluated in a larger patient cohort. Age is a determining factor that cannot be modified but needs to be considered when planning patients' therapy regimes. Our results show that patients above 70 correlate with higher *IL6* expression, which could be associated with less response to gemcitabine.

The general depletion of CAFs is proven to induce immunosuppression and accelerate pancreatic cancer with reduced survival [40].

In a broad TCGA cohort of patient data (*n* = 171) publicly available, we could confirm that a high *IL6* expression at the mRNA level is correlated with a significant reduction of disease-specific survival. However, this analysis cannot distinguish whether *IL6* derived from CAFs is responsible for this phenomenon. The RNA-seq was performed on bulk tissues, a mix of tumor and stromal cells. Further analysis using single-cell technologies may clarify this question.

An emerging subset in current fibroblast research are the apCAFs. So far, they have been identified in cholangiocarcinoma, pancreatic cancer, breast- and lung cancer [41–44].

Brekken et al. identified apCAFs deriving from mesothelial cells through TGFβ and IL-1 signaling [5]. They gain the function of inducing naïve CD4+ cells to become regulatory T-cells (Tregs) and support the tumor's immune evasion [5]. By use of an anti-Mesothelin Antibody (MSLN Ab), the group was able to show in vitro and in vivo significant reductions of dtTomato^+^ cells in TAM-induced WT1^CreERT2^; R26^LSL-tdTomato^ mice. Furthermore, they found a reduction of fibroblast features by α-SMA and IL6 [5]. In additional orthotopic mouse models, they confirmed that the use of MSLN Ab reduces tumor weights and reduced Tregs while increasing CD8^+^ T cells. These findings support the importance of further clinical investigations of apCAFs once more. In Brekken's study, they did not investigate any pre-cancer models or correlate their human tissues with any clinical parameters of patients.

Another study by Varveri et al. found single-cell transcriptome data enrichments in antigen processing and autophagy pathways of ${{\rm{\alpha }}}$SMA CAF clusters [45]. The group reduced Treg infiltration as an inflammatory reprogramming in CAFs via a conditional Atg5 knockout model in ${{\rm{\alpha }}}$SMA^+^ CAFs. Their study supports our observation that myCAF and iCAF features might overlap not just in expression but also in a functional manner.

Interestingly, we identified not only a very high population of apCAFs in PDAC (~40%), but we also discovered apCAFs in pancreatitis tissues. Our work complements the study from Elyada, Tuveson et al. and provides new information about the quantity of the MHC-II presenting CAFs. In 15 cancer tissues, the range of apCAF was between 13% and 79% with a median of 40%, double the amount of the previous primary study unrevealing this CAF group [19]. The reported quantitative expressions were ~20% apCAFs in human fibroblasts [5, 19]. DAFs in chronic pancreatitis patients also express MHC-II positive DAFs between 4 and 59%. In median, 20% of the DAFs express markers for apCAFs. However, additive investigations in a larger cohort are indispensable. Likewise, functional analysis of the DAF groups in the microenvironment of pancreatitis patients is essential.

Elyada, Tuveson et al. reported that apCAFs could present antigens to T-cells tested in MHCII-EGFP knock-in mice and an OTII mouse model. These fibroblast groups might provide targets limiting the genesis of life-threatening PDAC in the future. Studies assume already that stromal components/ cells act to restrain rather than support PDAC progression [46, 47].

Past investigations by Feig et al. assessed FAP^+^ CAFs as a principal source of the chemokine (C-X-C motif) ligand 12, which causes immune suppression in PDAC [48]. Upon depletion of FAP^+^ cells in the stroma, the antitumor activity of α-CTLA-4 and α-PD-L1 was potentiated. Due to our analysis, gender is respectively a trending factor because males express slightly higher levels of FAP and the myCAF marker *ACTA2*.

Recent studies interrogate CD74 as a marker for senescence in fibroblasts [49]. In our analysis, the expression of CD74 levels did not change even in higher cell culture passages of DAFs/CAFs. All samples expressing CD74 similarly express HLA receptors.

However, a limitation is that cultivated cells do not display the original in vivo microenvironment conditions. Ideally, spatial transcriptomics and single-cell analysis must be performed to characterize DAFs and CAFs further.

Further studies on functional relevance are needed to develop better 3D culturing matrix models, pancreatic cancer patient-derived organoids, and co-cultures [50].

The results of our study provide a new approach to classify the complexity and heterogeneity of fibroblasts in the microenvironment of pancreatic diseases. The discovery of similarities and differences between DAFs in chronic pancreatitis tissues and CAFs in cancer tissues is a novel finding for other studies to build up additional investigations on functional relevance.

Our study highlights the importance of individual cancer therapies for each patient depending on personalized risk factors.

Once the detailed functions of fibroblasts in the microenvironment are resolved, our study leads to the hypothesis that risk factor modification, like lifestyle changes, could influence the prevalence of specific CAF subsets. Patients under the age of 70 with a normal BMI, no diabetes, and smokers are respectively candidates for chemotherapy with gemcitabine due to low *IL6* expression.

However, further investigations are needed to determine which DAF and CAF subsets harbor tumor-promoting and tumor-restraining functions.

## Supplementary information

Supplementary information

## Data Availability

The datasets generated and/or analyzed during the current study are available from the corresponding author on reasonable request.
