# Supplementary material for: The influence of clinical risk factors on the classification of human cancer-associated fibroblasts in PDAC and pancreatitis patients
Source: BJC Rep. 2025 Jun 16;3:44. doi: 10.1038/s44276-025-00150-5 (PMC12170909; doi:10.1038/s44276-025-00150-5)
Supplement: Supplementary file 1 — Supplementary information [file 44276_2025_150_MOESM1_ESM.pdf]

Supplementary Figures for the paper:

The influence of clinical risk factors on the classification of human cancer-associated fibroblasts in PDAC patients.

Supplementary Figure 1

Gating strategy.

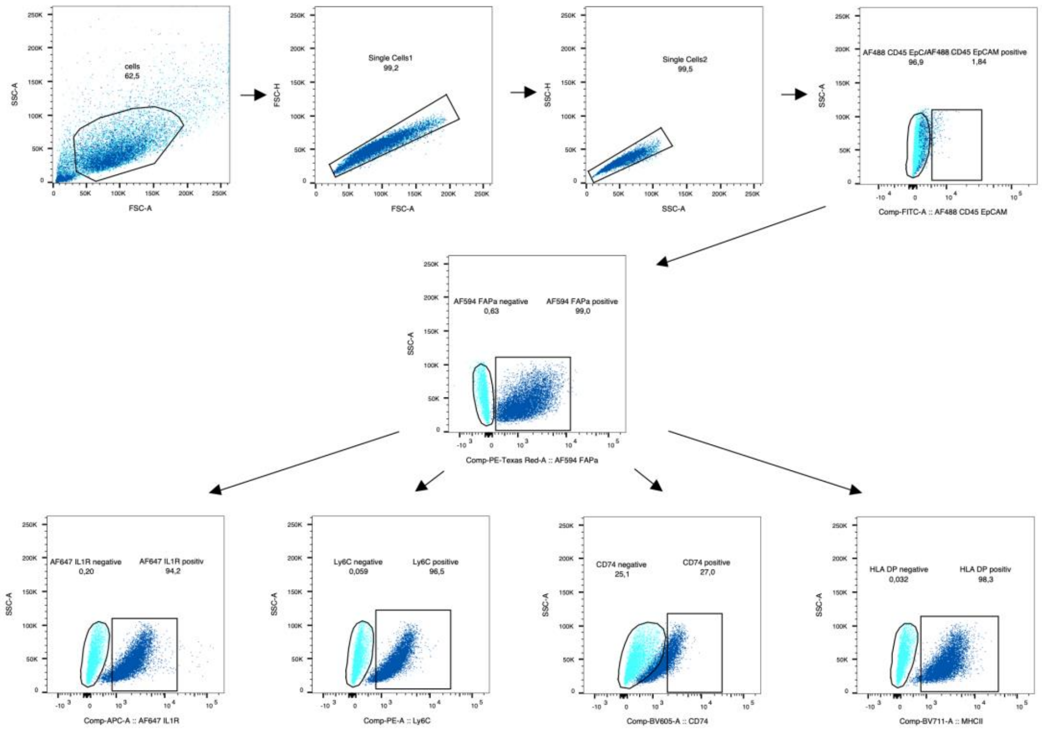

Supplementary Figure 2

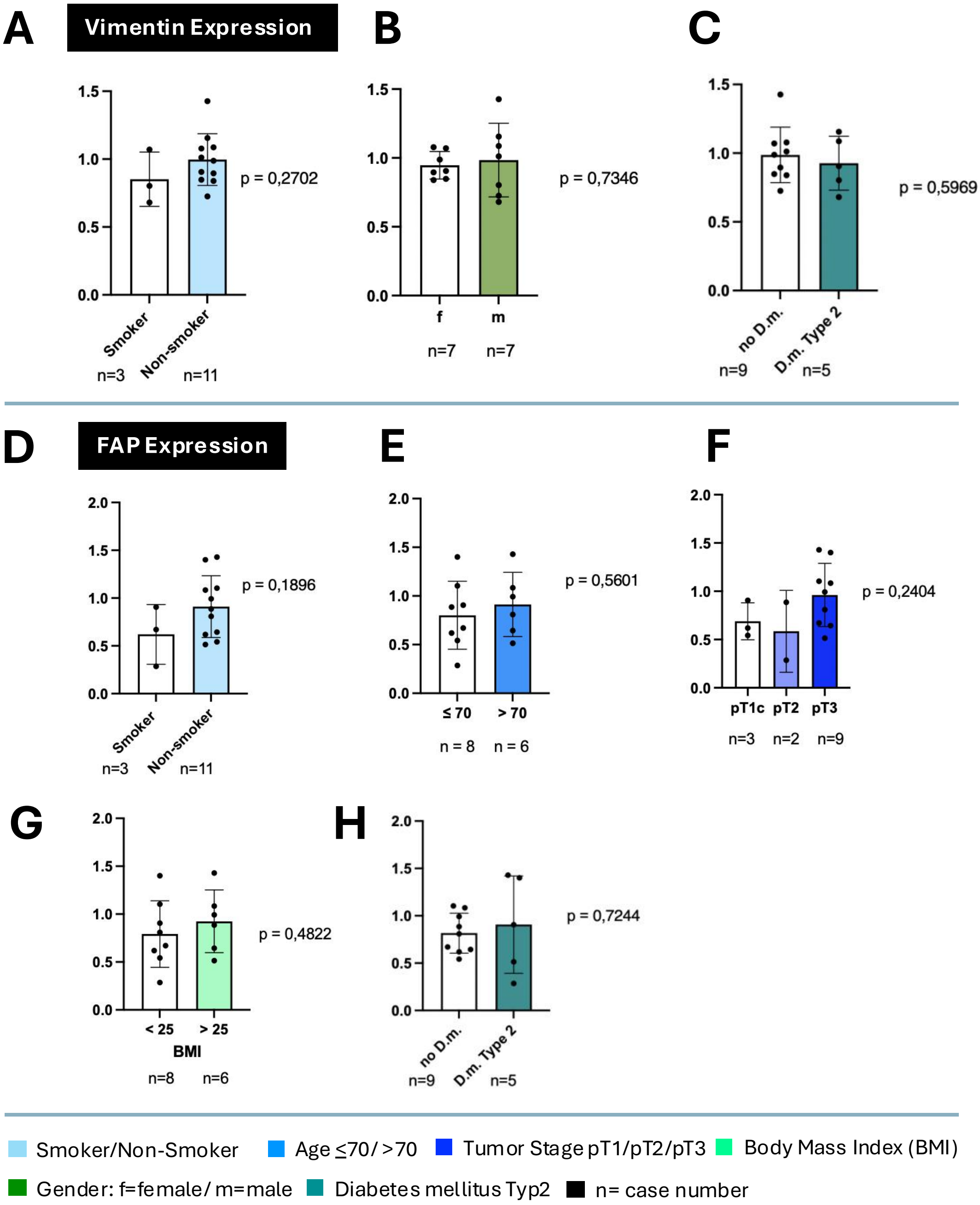

Supplementary Figure 3

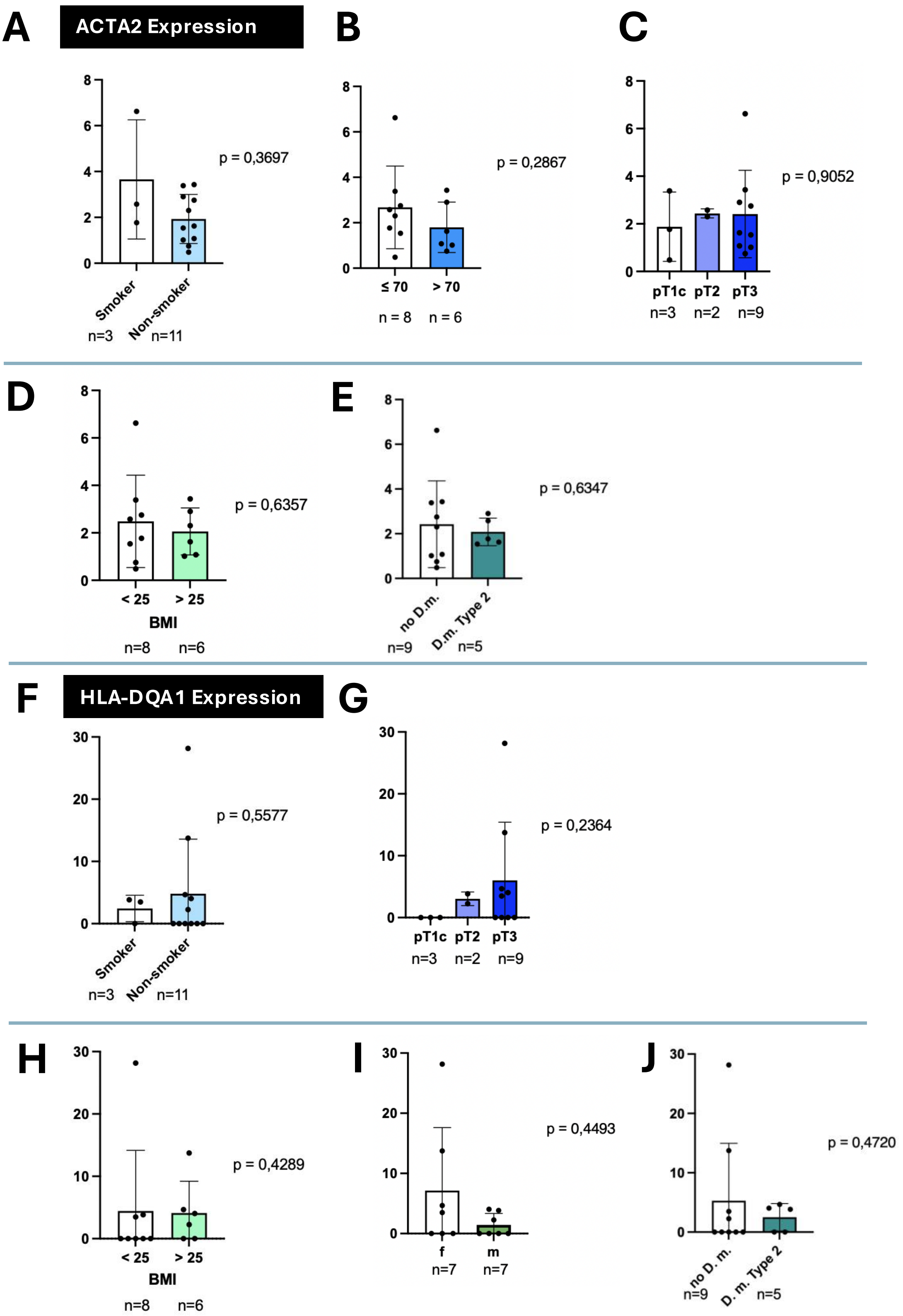

Smoker/Non-Smoker Age ≤70/ >70 Tumor Stage pT1/pT2/pT3 Body Mass Index (BMI)
Gender: f=female/ m=male Diabetes mellitus Typ2 n= case number

Supplementary Figure 4

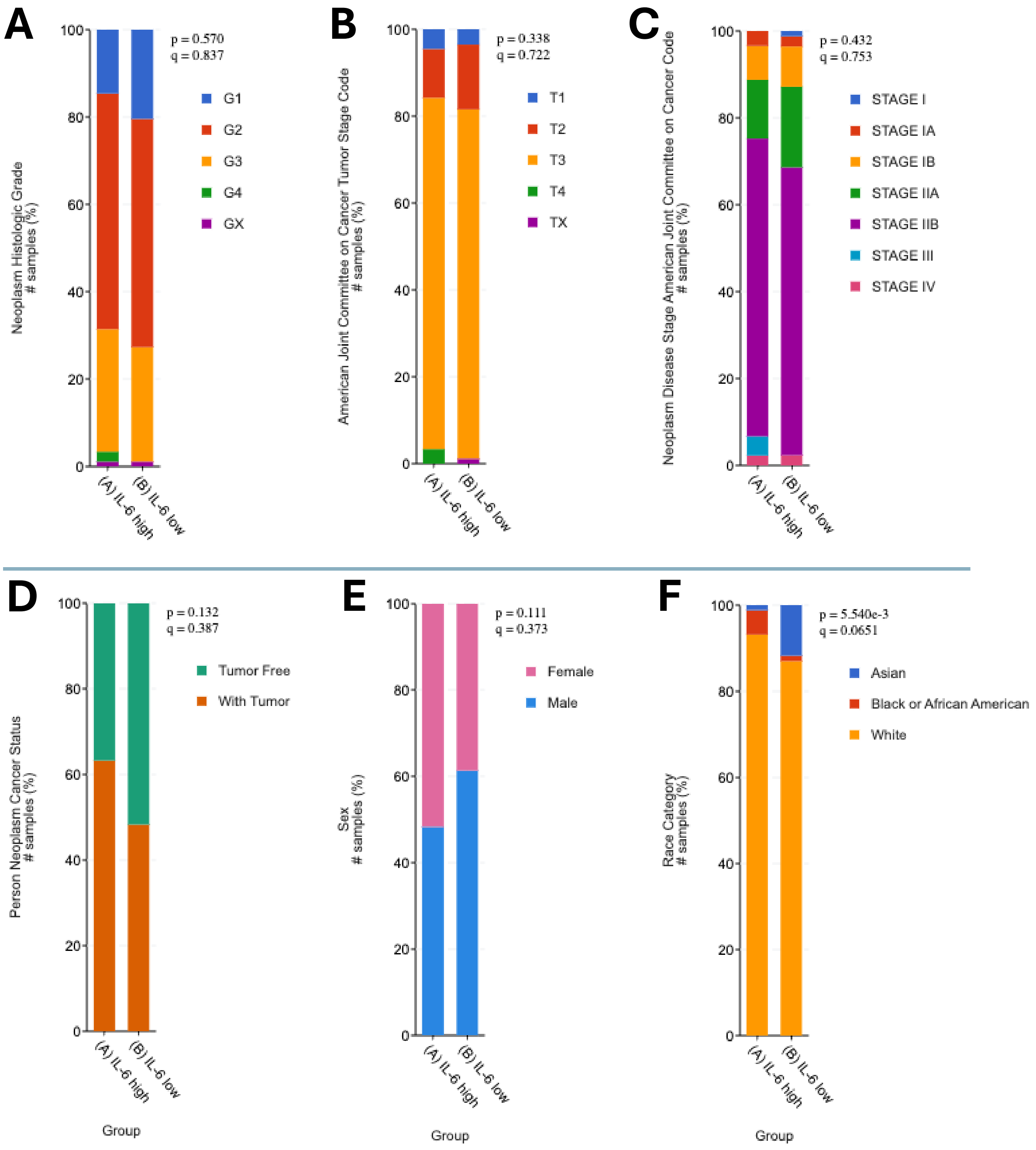

Supplementary Table 1

Neoadjuvant chemotherapy	Number of patients	Number of cycles
FOLFIRINOX (Leucovirin, Fluorouracil, Oxaliplatin, Irinotecan)	3	4,6,12
FOLFOXIRI (Leucovirin, Fluorouracil, Oxaliplatin, lower dose Irinotecan)	2	6,8
Nab-Paclitaxel/ Gemcitabin	1	3
